# OVER-TIME II trial: design and rationale of a multicentre randomized study evaluating antithrombotic strategies in patients with acute coronary syndrome and coronary artery ectasia

**DOI:** 10.1093/ehjopen/oeag161

**Published:** 2026-09-30

**Authors:** Edith Adame-Avilés, Diego Araiza-Garaygordobil, Lissette García-Mena, Marco Alcocer-Gamba, Ana Cristina Maldonado-May, Ricardo Álvarez-Santana, José Raúl Nieto-Saucedo, Alejandro Iván Rojas-Arriaga, Rodrigo Gopar-Nieto, Jorge Daniel Sierra-Lara Martínez, Luis Alfonso Marroquín-Donday, Jesús Díaz-Marín, Leonor Jacobo-Albavera, María Alexandra Arias-Mendoza, Ajit S Mullasari

**Affiliations:** Coronary Care Unit, Instituto Nacional de Cardiología Ignacio Chávez, Juan Badiano No. 1, Colonia Belisario Domínguez-Sección XVI, Tlalpan, 14080 Mexico City, Mexico; Plan of Combined Studies in Medicine (PECEM-MD/PhD), Faculty of Medicine, National Autonomous University of Mexico, Circuito Interior, Ciudad Universitaria, Coyoacán, 04510 Mexico City, Mexico; Coronary Care Unit, Instituto Nacional de Cardiología Ignacio Chávez, Juan Badiano No. 1, Colonia Belisario Domínguez-Sección XVI, Tlalpan, 14080 Mexico City, Mexico; Coronary Care Unit, Instituto Nacional de Cardiología Ignacio Chávez, Juan Badiano No. 1, Colonia Belisario Domínguez-Sección XVI, Tlalpan, 14080 Mexico City, Mexico; Centro de Estudios Clínicos Querétaro (CECLIQ), Querétaro 76000, Mexico; Coronary Care Unit, Instituto Nacional de Cardiología Ignacio Chávez, Juan Badiano No. 1, Colonia Belisario Domínguez-Sección XVI, Tlalpan, 14080 Mexico City, Mexico; Instituto Nacional de Cardiología Ignacio Chávez, Juan Badiano No. 1, Colonia Belisario Domínguez-Sección XVI, Tlalpan, 14080 Mexico City, Mexico; Instituto Nacional de Cardiología Ignacio Chávez, Juan Badiano No. 1, Colonia Belisario Domínguez-Sección XVI, Tlalpan, 14080 Mexico City, Mexico; Instituto Nacional de Cardiología Ignacio Chávez, Juan Badiano No. 1, Colonia Belisario Domínguez-Sección XVI, Tlalpan, 14080 Mexico City, Mexico; Coronary Care Unit, Instituto Nacional de Cardiología Ignacio Chávez, Juan Badiano No. 1, Colonia Belisario Domínguez-Sección XVI, Tlalpan, 14080 Mexico City, Mexico; Coronary Care Unit, Instituto Nacional de Cardiología Ignacio Chávez, Juan Badiano No. 1, Colonia Belisario Domínguez-Sección XVI, Tlalpan, 14080 Mexico City, Mexico; Department of Interventional Cardiology, Instituto Nacional de Cardiología Ignacio Chávez, Juan Badiano No. 1, Colonia Belisario Domínguez-Sección XVI, Tlalpan, 14080 Mexico City, Mexico; Department of Interventional Cardiology, Instituto Nacional de Cardiología Ignacio Chávez, Juan Badiano No. 1, Colonia Belisario Domínguez-Sección XVI, Tlalpan, 14080 Mexico City, Mexico; Laboratory of Cardiovascular Disease Genomics, National Institute of Genomic Medicine (INMEGEN), Periférico Sur No. 4809, Colonia Arenal Tepepan, Tlalpan, 14610 Mexico City, Mexico; Coronary Care Unit, Instituto Nacional de Cardiología Ignacio Chávez, Juan Badiano No. 1, Colonia Belisario Domínguez-Sección XVI, Tlalpan, 14080 Mexico City, Mexico; Madras Medical Mission, Mogappair, Chennai, 600037 Tamil Nadu, India

**Keywords:** Coronary artery ectasia, Antithrombotic therapy, Anticoagulation, Antiplatelet therapy, Genomic profiling

## Abstract

**Introduction:**

Coronary artery ectasia (CAE) is characterized by diffuse coronary dilatation and increased thrombotic risk. In patients with acute coronary syndrome (ACS) and CAE, the optimal antithrombotic strategy remains unclear. Previously, an exploratory trial suggested that the combination of antiplatelet monotherapy plus oral anticoagulation may reduce ischaemic events without increasing bleeding risk compared to dual antiplatelet therapy (DAPT), although it was underpowered for definitive conclusions.

**Aims:**

The aim of this study is to evaluate the efficacy and safety of antiplatelet monotherapy plus direct oral anticoagulation compared with DAPT in patients with ACS and culprit-vessel CAE in a multicentre randomized setting.

**Methods and results:**

OVER-TIME II is a multicentre, multinational, randomized, open-label clinical trial with 1:1 allocation comparing acetylsalicylic acid 100 mg plus clopidogrel 75 mg vs. clopidogrel 75 mg plus rivaroxaban 15 mg daily. The study will include 326 patients with ACS and culprit-vessel CAE. Patients will be followed for 12 months. The co-primary efficacy endpoint is the time to first occurrence of a composite of cardiovascular death, non-fatal myocardial infarction, or repeat revascularization. The co-primary safety endpoint is the time to first occurrence of major or minor bleeding defined by the Bleeding Academic Research Consortium criteria. Secondary endpoints include individual components of the primary outcomes. Exploratory analyses will include genomic and transcriptomic profiling through DNA and RNA sampling to identify molecular signatures associated with thrombotic risk, treatment response, and clinical outcomes.

**Conclusion:**

OVER-TIME II is designed to provide robust multicentre randomized evidence regarding the efficacy and safety of an anticoagulation-based strategy in patients with ACS and CAE, with the potential to contribute to future clinical research and guideline development.

**Registration:**

NCT07511257. The trial is registered and publicly available on ClinicalTrials.gov and is currently actively recruiting patients.

## Graphical Abstract

**Graphical Abstract:**
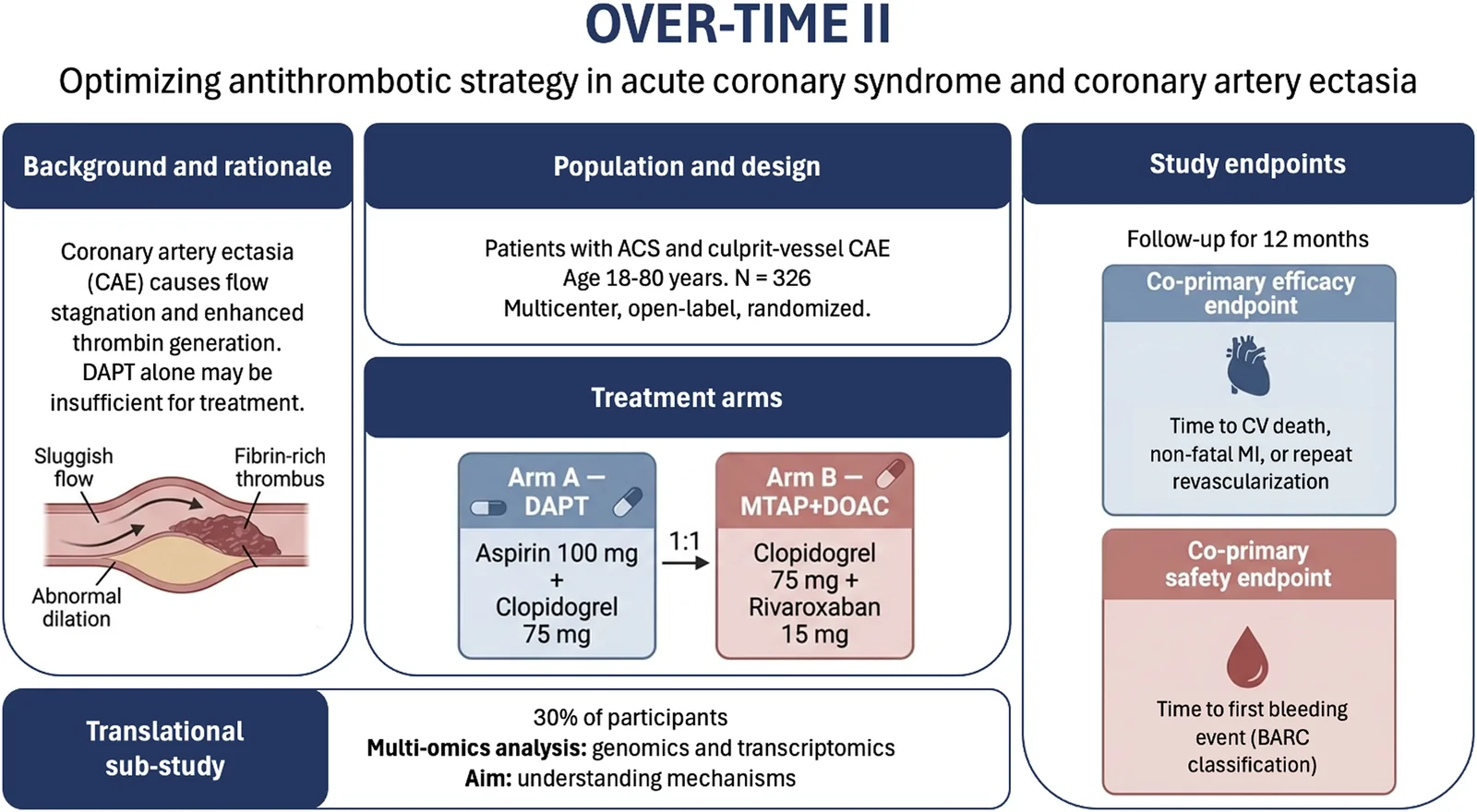

## Introduction

Coronary artery ectasia (CAE) is defined as an abnormal dilatation of a coronary artery segment exceeding 1.5 times the diameter of an adjacent normal segment.^1,2^ Its prevalence ranges from 0.3 to 4.9%,^1,3^ with higher rates described in specific populations, including Mexico, where CAE has been reported in up to 10.3% of patients presenting with ST-elevation myocardial infarction.^4^

Coronary artery ectasia has been associated with altered coronary flow dynamics, including flow stagnation and turbulence, which promote thrombus formation and could explain the increased rates of ischaemic events.^5–8^ In addition to these haemodynamic alterations, patients with CAE exhibit a prothrombotic and inflammatory phenotype characterized by increased platelet reactivity, endothelial dysfunction, and enhanced thrombin generation.^5,9,10^ Platelet activation is reflected by higher P-selectin levels, while slow coronary flow promotes local thrombin and fibrin formation.^10,11^ Emerging evidence also suggests the involvement of genetic and post-transcriptional regulatory mechanisms, including alterations in gene expression and microRNA profiles^12–14^. As a result, patients with CAE and acute coronary syndrome (ACS) represent a high-risk subgroup with increased rates of ischaemic events.^6,7^

Despite these pathophysiological considerations, optimal antithrombotic management in patients with ACS and CAE remains undefined. Current treatment strategies are largely extrapolated from broader ACS populations, with dual antiplatelet therapy (DAPT) representing the standard of care.^15–17^ However, the distinct prothrombotic milieu associated with CAE provides a strong pathophysiological rationale for combined anticoagulation–antiplatelet therapy, targeting both platelet activation and coagulation pathways involved in thrombin generation and fibrin formation. Anticoagulation is frequently used in clinical practice among patients with CAE, although its benefit remains uncertain.^6,16^ Observational studies and small case series suggest a potential reduction in recurrent ischaemic events with anticoagulation, particularly when therapeutic levels are consistently achieved,^6,16,18^ whereas larger cohort studies and meta-analyses have shown inconsistent results, with no clear superiority over DAPT.^19,20^

To address this gap, the OVER-TIME trial was conducted as the first randomized study evaluating antithrombotic strategies in patients with ACS and CAE.^21^ This exploratory trial compared standard DAPT with a strategy combining antiplatelet monotherapy and direct oral anticoagulation (DOAC), demonstrating a numerical reduction in major adverse cardiovascular events, decreased recurrence of ischaemic events, and a significant reduction in fibrin clot lysis time in the anticoagulation group, without a corresponding increase in bleeding events.^22^ However, the study was limited by its sample size and exploratory design and therefore was not powered to detect statistically significant differences in clinical outcomes.

These findings provide a strong biological and clinical rationale for further investigation in a larger, adequately powered randomized trial. OVER-TIME II has been designed as a multicentre, multinational, randomized clinical trial to evaluate the efficacy and safety of an anticoagulation-based strategy (clopidogrel 75 mg plus rivaroxaban 15 mg daily) compared with standard DAPT (acetylsalicylic acid 100 mg plus clopidogrel 75 mg daily) in patients with ACS and CAE.

The rivaroxaban dose of 15 mg daily was intentionally selected to maintain consistency with the exploratory OVER-TIME trial, allowing comparability between studies and building upon the preliminary clinical safety data generated with this regimen. This dose provides sustained factor Xa inhibition while representing a reduced-dose strategy compared with the standard 20 mg regimen, balancing antithrombotic effect with bleeding risk. Among direct oral anticoagulants, rivaroxaban has the most extensive prospective evidence supporting reduced-dose regimens in combination with antiplatelet therapy in a randomized clinical trial setting, with this strategy demonstrating reduced bleeding risk without an apparent increase in ischaemic events.^23,24^

In addition to its clinical objectives, the study incorporates a translational component aimed at elucidating the molecular mechanisms underlying thrombotic risk in CAE. Preliminary data suggest differential expression of circulating microRNAs and the presence of genetic variants in patients with CAE, supporting the hypothesis that molecular alterations may contribute to disease phenotype and thrombotic susceptibility.^13,25,26^ Therefore, a predefined translational sub-study will be conducted as a discovery-phase investigation in a subset of patients (≈30% of the study population). The primary biological hypothesis is that patients with CAE will exhibit distinct genomic and transcriptomic profiles involving pathways related to angiogenesis, vascular inflammation, extracellular matrix remodelling, endothelial dysfunction, platelet activation, coagulation, and pharmacogenomic response to antithrombotic therapy and that these molecular signatures may differ according to treatment strategy and clinical outcomes. Given the anticipated sample size, all genomic and transcriptomic findings will be considered hypothesis generating rather than confirmatory (*Graphical abstract*).

The present study aims to evaluate the efficacy and safety of an anticoagulation-based strategy compared with standard DAPT in patients with ACS and CAE while also providing mechanistic insights into the biology of CAE.

## Methods

### Study design and population

OVER-TIME II is a multicentre, multinational, randomized, open-label clinical trial designed to evaluate antithrombotic strategies in patients with ACS and CAE. A total of 326 patients will be enrolled across participating centres, with an anticipated mean follow-up of at least 12 months (*Figure 1*).

**Figure 1:**
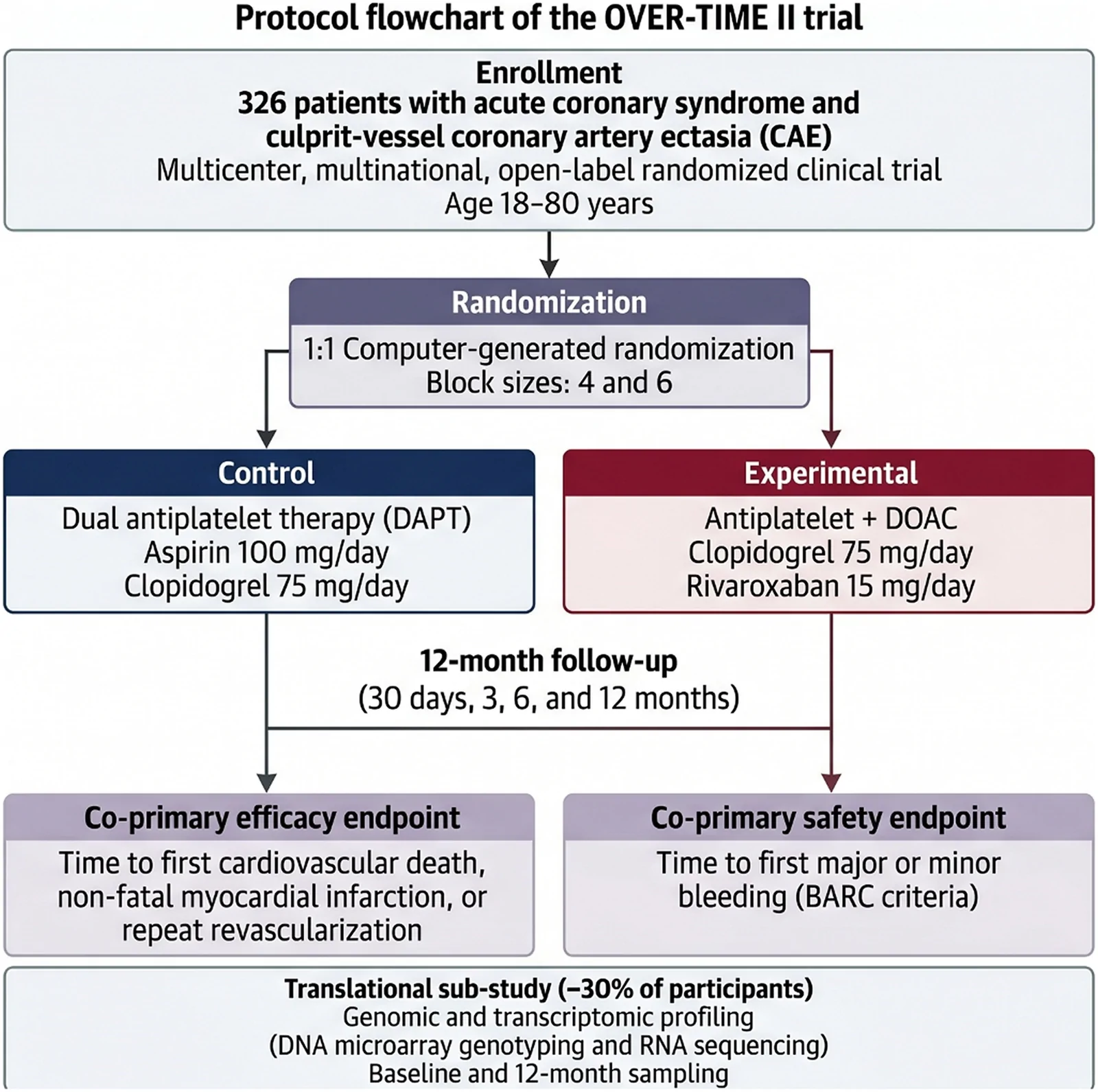
Protocol flowchart of the OVER-TIME II trial.

Patients aged 18–80 years presenting with ACS (ST-elevation or non-ST-elevation myocardial infarction) and angiographically confirmed CAE in the culprit vessel are eligible for inclusion. Coronary artery ectasia is defined as a coronary artery dilatation exceeding 1.5 times the diameter of an adjacent normal segment, as assessed by two independent interventional cardiologists and confirmed by quantitative coronary angiography (QCA) in a blinded core laboratory. Key exclusion criteria include indication for the use of anticoagulation, prior bleeding risk according to investigator criteria, severe renal impairment (estimated glomerular filtration rate < 30 mL/min/1.73 m^2^), recent major bleeding events, and advanced, refractory heart failure (*Table 1*).

**Table 1:** Eligibility criteria for OVER-TIME II

**Inclusion criteria**
1. Age 18–80 years, hospitalized with acute coronary syndrome, with or without ST-segment elevation 2. Angiographically confirmed coronary artery ectasia in the culprit vessel and one- or two-vessel coronary artery disease, defined as: Coronary dilatation ≥ 1.5 times the diameter of an adjacent normal segment Adjudicated by two independent interventional cardiologists and confirmed by quantitative coronary angiography 3. Culprit vessel identified based on electrocardiographic territory and/or angiographic evidence of atherothrombosis [e.g. thrombus or reduced flow] 4. Hospital stay > 24 h 5. Percutaneous coronary intervention or thrombolytic management, as deemed appropriate by the treating team 6. Provision of written informed consent
**Exclusion criteria**
1. Pregnancy 2. Indication for temporary or permanent anticoagulation at the time of enrolment 3. Severe chronic kidney disease (KDIGO Stage IV or higher, or eGFR < 30 mL/min/1.73 m^2^) 4. Estimated glomerular filtration rate < 30 mL/min/1.73 m^2^ at hospital discharge 5. History of major bleeding (e.g. gastrointestinal bleeding and intracranial haemorrhage) or high bleeding risk 6. Advanced heart failure, defined as left ventricular ejection fraction < 30% plus at least one of the following: ≥2 hospitalizations or unplanned emergency visits within the past year NYHA Class III or higher despite optimal medical therapy within the last 3 months

NYHA: New York Heart Association; eGFR: Estimated Glomerular Filtration Rate; KDIGO: Kidney Disease: Improving Global Outcomes.

### Randomization and treatment allocation

Participants will be randomized in a 1:1 ratio to receive either DAPT or antiplatelet monotherapy plus DOAC. The randomization sequence was generated before study initiation using a computer-generated algorithm in R (R Foundation for Statistical Computing) with randomly varying block sizes of 4 and 6 to maintain treatment balance while minimizing allocation predictability.

The sequence was generated by an investigator who was not involved in participant recruitment, enrolment, or treatment allocation. The randomization list was stored in a blinded electronic file and is not accessible to investigators responsible for patient recruitment. Treatment assignment was provided by an independent third party only after confirmation of eligibility and completion of informed consent.

Given the nature of the interventions, complete allocation concealment from patients and investigators is not feasible; therefore, the study will be conducted in an open-label fashion.

### Study procedures and follow-up

Patients will be recruited during hospitalization and randomized after confirmation of eligibility criteria and provision of informed consent. Clinical follow-up will be conducted at 30 days and 12 months, with additional telephone contacts at 3 and 6 months, all performed by an investigator blinded to treatment allocation. At each time point, clinical status, treatment adherence, concomitant medications (including drugs with potential interactions with rivaroxaban), and occurrence of predefined endpoints will be assessed and documented.

Every effort will be made to obtain complete follow-up data for all randomized participants. Patients who cannot be reached during scheduled follow-up visits will undergo repeated contact attempts through telephone calls. Patients will be considered lost to follow-up after failure to complete two consecutive scheduled follow-up assessments despite repeated contact attempts. All available clinical information obtained before loss to follow-up will be retained for analysis.

### Study endpoints

The trial has two co-primary endpoints. The primary efficacy endpoint is defined as the time to first occurrence of a composite of cardiovascular death, non-fatal myocardial infarction, or repeat coronary revascularization. Definitions of cardiovascular death and repeat coronary revascularization will follow the Academic Research Consortium-2 consensus, while myocardial infarction will be defined according to the Fourth Universal Definition of Myocardial Infarction.

The primary safety endpoint is defined as the time to first occurrence of bleeding events classified according to the Bleeding Academic Research Consortium (BARC) classification.

Secondary endpoints include the individual components of the co-primary efficacy endpoint (cardiovascular death, non-fatal myocardial infarction, and repeat coronary revascularization); the individual components of the primary safety endpoint, classified according to BARC criteria; and all-cause mortality.

### Blinding and bias mitigation

To minimize performance and detection bias, the investigators responsible for patient follow-up will be blinded to treatment allocation. Additionally, all suspected endpoint events will be adjudicated by an independent CEC. Standardized definitions for all efficacy and safety endpoints will be applied uniformly across all participating centres, as described in the Study endpoints section. Participants will receive standardized counselling regarding medication adherence at each follow-up visit, and adherence will be assessed using the Simplified Medication Adherence Questionnaire (SMAQ). Investigators at each site will receive standardized training regarding study procedures and endpoint assessment.

### Sample size calculation

The sample size was calculated based on the primary efficacy endpoint (the time to first occurrence of the composite of cardiovascular death, non-fatal myocardial infarction, or repeat coronary revascularization). As the OVER-TIME trial is the only randomized trial evaluating antithrombotic strategies in patients with ACS and CAE, it represents the best available prospective evidence in this population. Based on that study, event-free survival was assumed to be 87.5% in the DAPT group and 96.7% in the antiplatelet monotherapy plus DOAC group. Under these assumptions, 326 patients (163 per group) provide 80% power to detect the anticipated treatment effect using a two-sided log-rank test with an alpha level of 0.05. The calculation allowed for an anticipated crossover rate of up to 5%.

To assess the uncertainty of the sample size assumptions, sensitivity analyses were performed considering alternative event-rate scenarios reflecting a range of plausible treatment effects (see Supplementary material online, *Table S1*). Across the alternative scenarios evaluated, the required sample size ranged from 398 to 763 patients. These findings illustrate the dependence of the required sample size on the assumed treatment effect and acknowledge the uncertainty inherent in planning a trial in a population with limited prior randomized evidence. Accordingly, the protocol includes two pre-specified interim futility analyses at ∼25% (*n* = 82) and 50% (*n* = 163) of the planned enrolment to evaluate the conditional power of the trial and determine whether continuation of the study remains justified under the observed event rates and treatment effect.

### Statistical analysis

Continuous variables will be reported as mean ± standard deviation or median (interquartile range), as appropriate according to data distribution. Categorical variables will be expressed as counts and percentages. Baseline characteristics will be compared using the χ^2^ test or Fisher’s exact test for categorical variables, and Student’s *t*-test or the Wilcoxon rank-sum test for continuous variables, as appropriate.

The co-primary endpoints will be analysed as time-to-first-event outcomes. The primary efficacy analysis will be conducted according to the intention-to-treat principle, whereby all randomized participants will be analysed according to their assigned treatment group regardless of treatment adherence, treatment crossover, or protocol deviations. In addition, a per-protocol analysis excluding participants with major protocol deviations will be performed as a sensitivity analysis. Event-free survival will be estimated using the Kaplan–Meier method and compared using the log-rank test. Hazard ratios and 95% confidence intervals will be estimated using Cox proportional hazards models. Secondary and exploratory endpoints will be analysed using appropriate statistical tests depending on variable type and distribution. For analyses of individual non-fatal endpoints, competing risk methods (Fine and Gray subdistribution hazard models) will be considered when appropriate.

All statistical analyses will be performed using R software (R Foundation for Statistical Computing, Vienna, Austria). A two-sided *P* value of <0.05 will be considered statistically significant. Two pre-specified interim analyses are planned: the first after enrolment of 25% of the target sample (≈82 patients) and the second after enrolment of 50% (≈163 patients). Both interim analyses will focus on futility assessment using the Lan–DeMets spending function with O’Brien–Fleming boundaries. If the conditional power falls below 20% at interim, the trial may be stopped for futility. No efficacy stopping rules are pre-specified at the interim analyses. Final significance thresholds for the primary endpoints will be preserved at the two-sided alpha level of 0.05.

### Trial oversight and safety monitoring

The study will be overseen by a Steering Committee composed of the principal investigators from the coordinating centre and participating sites. The Steering Committee will be responsible for overall study conduct, recruitment oversight, protocol adherence, safety, data quality, and review of interim analyses. All suspected endpoint events will be independently adjudicated by a blinded Clinical Events Committee (CEC), whose members are not involved in patient recruitment or treatment and are blinded to treatment allocation. The CEC will adjudicate efficacy and safety endpoints according to pre-specified standardized definitions.

Participant safety will be continuously monitored throughout the study. Suspected bleeding events will be reported by the participating investigators and subsequently adjudicated by the CEC, whose members are blinded to treatment allocation. Safety assessments will include bleeding events classified according to the BARC criteria, all-cause and cardiovascular mortality, and other serious adverse events of potential relevance to the study interventions.

Study treatment may be temporarily interrupted or permanently discontinued in an individual participant in the event of BARC Type 5 bleeding, intracranial haemorrhage, life-threatening or major bleeding requiring urgent intervention or transfusion, or any serious adverse event considered by the treating investigator to represent an unacceptable risk associated with continued study treatment. Individual treatment discontinuation will not result in withdrawal from study follow-up, and participants will continue to be followed according to the study protocol whenever possible.

The Steering Committee may request an unscheduled safety review at any time if a clinically important safety concern arises. A formal safety signal will be considered if any of the following are observed in either treatment group: (i) an unexpected excess of adjudicated BARC Type 3–5 bleeding; (ii) two or more adjudicated intracranial haemorrhages or fatal bleeding events; (iii) an unexpected excess of all-cause or cardiovascular mortality; (iv) an unexpected excess of major adverse cardiovascular events, including myocardial infarction, coronary revascularization, or cardiovascular death; or (v) an accumulation of serious adverse events considered related to study treatment that may alter the overall benefit–risk assessment.

These thresholds will trigger a formal review and will not constitute automatic termination of the trial. The Steering Committee will consider the number, severity, timing, treatment relatedness, and distribution of events between treatment groups, together with the accumulating efficacy data, before recommending continuation, enhanced safety monitoring, temporary suspension of recruitment or treatment allocation, protocol modification, or early termination of the trial.

### Exploratory and mechanistic analyses

The translational sub-study will be conducted in ≈30% of the study population (*Figure 2*). Peripheral blood samples will be collected at randomization and at 12-month follow-up for genomic and transcriptomic profiling. A subpopulation of 30 patients with CAE will be analysed before and after DAPT therapy, and 30 samples will be analysed before and after MTAP + DOAC therapy, for a total of 60 CAE samples. In addition, 20 control samples with SCA will also be analysed.

**Figure 2:**
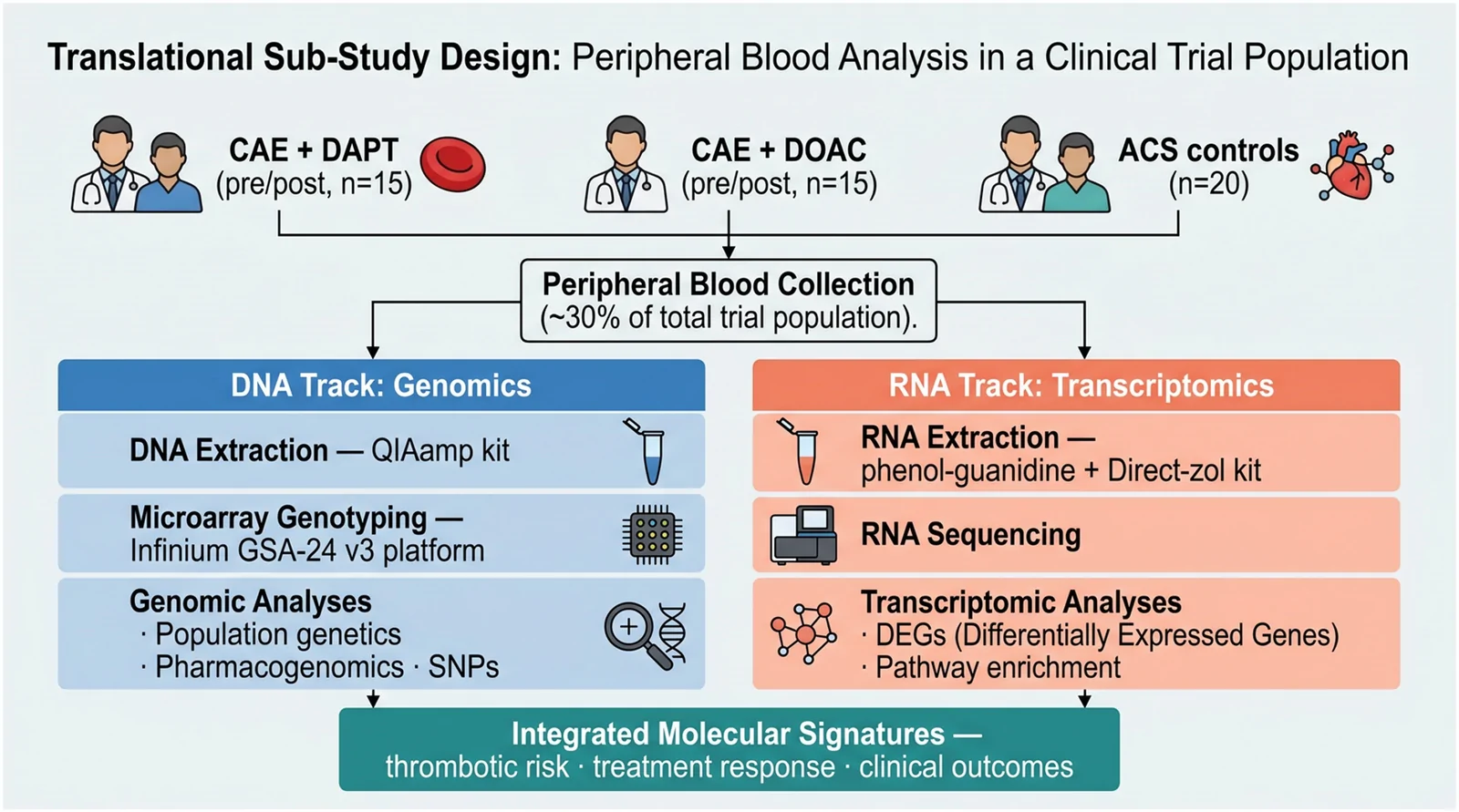
Design of the translational sub-study. Peripheral blood samples will be collected at baseline and 12-month follow-up in ∼30% of trial participants (*n* = 98), including patients with CAE receiving DAPT, CAE receiving DOAC plus antiplatelet monotherapy, and ACS controls without CAE. Samples undergo parallel genomic (microarray genotyping, pharmacogenomics, SNP profiling) and transcriptomic (RNA sequencing, differential gene expression, pathway enrichment) analyses. Integrated findings aim to identify molecular signatures associated with thrombotic risk, treatment response, and clinical outcomes. This is a discovery-phase, hypothesis-generating sub-study. CAE, coronary artery ectasia; DAPT, dual antiplatelet therapy; DOAC, direct oral anticoagulant; SNPs, single nucleotide polymorphisms.

For genomic analyses, DNA extraction will be performed using the QIAamp DNA Blood Midi Kit (QIAGEN). The microarrays used will be Infinium Global Screening Array-24 v3, and the analysis will be conducted at the INMEGEN Microarray Unit. These microarrays contain single nucleotide polymorphisms (SNPs) that support population genetics analysis, pharmacogenomic studies, and precision medicine research.

For transcriptomic analyses, RNA extraction will be performed using the phenol–guanidine isothiocyanate method, followed by column-based extraction with the Direct-zol RNA MiniPrep Kit (Zymo Research). Transcriptomic analysis will be performed using RNA-seq at the INMEGEN Sequencing Unit. The RNA-seq data will undergo quality assessment, read filtering, alignment to the reference genome, transcript quantification, normalization, differential gene expression analysis, and pathway enrichment analyses using established bioinformatic pipelines. To account for multiple testing, differential expression analyses will control the false discovery rate using the Benjamini–Hochberg procedure.

The translational analyses are intended to generate biological hypotheses rather than identify clinically applicable biomarkers. Specifically, they will explore differentially expressed genes between CAE and SCA; characterize altered molecular pathways related to vascular inflammation, arterial remodelling, endothelial dysfunction, platelet activation, and thrombosis; and identify transcriptomic signatures associated with the co-primary clinical endpoints and response to antithrombotic therapy. Any candidate molecular signatures identified in this discovery-phase sub-study will require validation in independent cohorts before potential clinical application.

### Ethical considerations

The study will be conducted in accordance with the Declaration of Helsinki, Good Clinical Practice guidelines, and applicable national and international regulations. The protocol and any subsequent amendments will be approved by the appropriate institutional research ethics committees and regulatory authorities before implementation. Written informed consent will be obtained from all participants before any study-specific procedure.

## Discussion

OVER-TIME II is designed as a multicentre, open-label, randomized clinical trial that aims to evaluate antithrombotic strategies in patients with ACS and CAE. Given the absence of randomized evidence in this population, the optimal antithrombotic strategy remains uncertain. Building upon the signals observed in the exploratory OVER-TIME trial,^22^ OVER-TIME II seeks to address this gap by evaluating whether an anticoagulation-based strategy combining clopidogrel and rivaroxaban improves clinical outcomes while maintaining an acceptable safety profile compared with standard DAPT. If successful, the findings of this study may help inform future research and clinical decision-making regarding antithrombotic treatment in this high-risk population.

Coronary artery ectasia is characterized by abnormal coronary dilatation associated with altered haemodynamics and a prothrombotic state.^6,10,27^ The mechanistic rationale for anticipating a benefit of anticoagulation-based therapy over DAPT in this population is rooted in the pathophysiology of thrombus formation in CAE, which involves both platelet activation and enhanced thrombin generation. Accordingly, targeting both pathways through combined anticoagulant–antiplatelet therapy may provide more effective thrombotic protection than DAPT alone, without increasing the bleeding risk. Consistent with this hypothesis, the exploratory OVER-TIME trial demonstrated a numerical reduction in ischaemic events with a significant reduction in endogenous fibrin clot lysis time in patients receiving the anticoagulation-based strategy.^22^

Evidence guiding antithrombotic therapy in CAE remains scarce and is largely inconsistent.^6,16,18,19^ The OVER-TIME trial provided the first randomized evidence in this field, demonstrating a tendency towards reduced major adverse cardiovascular events and improved fibrinolytic profiles with an anticoagulation-based strategy, without a significant increase in bleeding.^22^ Nonetheless, its limited sample size and exploratory design need confirmation in a larger, adequately powered setting. OVER-TIME II will incorporate several methodological features that strengthen its potential to provide definitive evidence. The multicentre design enhances external validity and generalizability across diverse clinical settings.

Importantly, the study integrates a predefined translational sub-study in a subset of ≈30% of participants, including genomic and transcriptomic profiling. This component may provide novel mechanistic insights into the molecular pathways underlying thrombotic risk and variability in treatment response, potentially paving the way for precision-based therapeutic approaches in CAE.

If the hypothesis is confirmed, OVER-TIME II will provide randomized evidence regarding antithrombotic therapy in patients with ACS and CAE and may contribute to future clinical practice and guideline development, pending confirmation in independent studies. Moreover, the exploratory molecular profiling sub-study may generate biological hypotheses and identify candidate molecular signatures associated with treatment response, providing a foundation for future precision medicine research in patients with CAE.

### Study limitations

Several limitations and contextual considerations deserve acknowledgement. First, the open-label design carries an inherent risk of performance and detection bias. However, this risk is mitigated by an independent, blinded CEC adjudication of all primary endpoint events, standardized endpoint definitions applied uniformly across sites, and prospective adherence monitoring. A per-protocol sensitivity analysis will further complement the intention-to-treat primary analysis.

Second, the sample size assumptions were derived from the exploratory OVER-TIME trial, which was conducted at a single centre with a limited number of events. Consequently, if the true event rates differ materially from those anticipated, the study may be under- or overpowered, underscoring the importance of the pre-specified interim futility analyses.

Third, the eligibility criteria of OVER-TIME II, while appropriate to ensure internal validity and patient safety, limit direct generalizability of the findings to patients requiring chronic anticoagulation, with severe renal dysfunction, advanced heart failure, or high bleeding risk. Therefore, the results should be interpreted within the context of patients with culprit-vessel CAE presenting with ACS who meet these criteria. Future pragmatic studies and real-world registries will be needed to evaluate antithrombotic strategies in broader CAE populations with a greater burden of comorbidities.

Fourth, although OVER-TIME II focuses on clinical efficacy and safety endpoints, future studies should incorporate patient-centred outcomes, including health-related quality of life, functional recovery, recurrent angina, healthcare utilization, and cost-effectiveness, to provide a more comprehensive assessment of the overall value of antithrombotic strategies in patients with CAE.

Finally, the translational sub-study is designed as a discovery-phase investigation, and its molecular findings must be interpreted as exploratory and hypothesis generating. Any candidate molecular signatures identified will require independent validation in external cohorts before potential application.

Collectively, these considerations highlight that OVER-TIME II is intended to address an important evidence gap in the management of ACS with CAE while providing a foundation for future confirmatory, pragmatic, and translational studies in this understudied population.

## Conclusions

OVER-TIME II is designed to provide the first multicentre randomized evidence to address the central clinical question of optimal antithrombotic therapy in patients with ACS and CAE. The findings are expected to provide high-quality evidence regarding the efficacy and safety of this approach while advancing the biological understanding of CAE through an integrated translational sub-study.

## Lead author biography


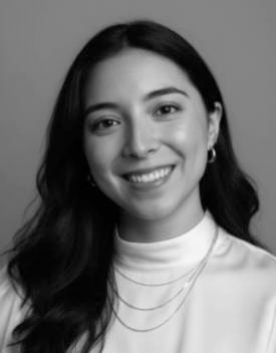
Dr Edith Adame is an early-career physician, clinical research coordinator, and investigator from Mexico with research interests in ACSs, CAE, and STEMI systems of care. She has conducted research at the National Institute of Cardiology Ignacio Chávez, the National Institute of Medical Sciences and Nutrition Salvador Zubirán, and Vanderbilt University Medical Center. Her work focuses on cardiovascular outcomes research and multinational clinical research collaborations in interventional cardiology.

## Supplementary Material

oeag161_Supplementary_Data

## Data Availability

The data underlying this article will be shared on reasonable request to the corresponding author.
