# Supplementary material for: OVER-TIME II trial: design and rationale of a multicentre randomized study evaluating antithrombotic strategies in patients with acute coronary syndrome and coronary artery ectasia
Source: Eur Heart J Open. 2026 Sep 30;6(6):oeag161. doi: 10.1093/ehjopen/oeag161 (PMC13728344; doi:10.1093/ehjopen/oeag161)
Supplement: Supplementary file 1 [file oeag161_supplementary_data.docx]

**Supplementary Table S1.** Sensitivity analysis of sample size requirements under alternative event-rate assumptions

|  | Scenario | 12-month event-free rate DAPT | 12-month event-free rate MTAP+DOAC | ARR (pp) | RR | N per arm | N total | Notes |
| --- | --- | --- | --- | --- | --- | --- | --- | --- |
| Pri | **Primary assumption (OVER-TIME I data)** | **87.5%** | **96.7%** | **9.2** | **0.264** | **163** | **326** | *Event rates derived from OVER-TIME I (Araiza-Garaygordobil et al., 2021)* |
| 1 | Scenario 1 — Slightly reduced experimental arm benefit | 87.5% | 96.0% | 8.5 | 0.320 | 199 | 398 | *MTAP+DOAC event-free rate reduced by 0.7 pp; DAPT rate unchanged* |
| 2 | Scenario 2 — Moderate benefit, same DAPT event rate | 87.5% | 95.0% | 7.5 | 0.400 | 271 | 542 | *MTAP+DOAC event-free rate reduced to 95.0%; DAPT rate unchanged* |
| 3 | Scenario 3 — Smaller benefit, same DAPT event rate | 87.5% | 94.0% | 6.5 | 0.480 | 382 | 763 | *MTAP+DOAC event-free rate reduced to 94.0%; DAPT rate unchanged* |
| 4 | Scenario 4 — Higher true event rate in DAPT arm (A) | 86.0% | 95.0% | 9.0 | 0.357 | 205 | 409 | *Higher baseline event rate in DAPT; ARR ~9 pp preserved* |
| 5 | Scenario 5 — Higher event rates, both arms (A) | 85.0% | 94.0% | 9.0 | 0.400 | 226 | 451 | *Both arms shifted to higher event rates; absolute reduction maintained* |
| 6 | Scenario 6 — Higher event rates, both arms (B) | 84.0% | 93.0% | 9.0 | 0.437 | 248 | 495 | *Further inflation of event rates; ARR maintained at 9 pp* |
| 7 | Scenario 7 — Pessimistic (A) | 83.0% | 92.0% | 9.0 | 0.471 | 269 | 538 | *Materially higher event rates than observed in OVER-TIME I* |
| 8 | Scenario 8 — Pessimistic (B) | 82.0% | 91.0% | 9.0 | 0.500 | 291 | 581 | *Worst-case scenario; 12-month event-free rate 82% in DAPT arm* |

*ARR = absolute reduction in 12-month event rate (DAPT − MTAP+DOAC), expressed as percentage points (pp). RR = relative risk of event in MTAP+DOAC versus DAPT arm. N per arm and N total account for a 5% crossover rate using the inflation factor ⌈N⌉/(1−crossover)², following the two-step ceiling procedure that reproduces the pre-specified N=326.*

*All scenarios: two-sided α=0.05, 80% statistical power, 1:1 allocation, 5% crossover. Formula: Freedman (1982) log-rank sample size, E = (z_α/2_ + z_β_)² × (1+RR)² / (1−RR)²; N = E / p̄, where p̄ = (e₁+e₂)/2.*

*DAPT, dual antiplatelet therapy (aspirin 100 mg + clopidogrel 75 mg); MTAP+DOAC, antiplatelet monotherapy plus direct oral anticoagulation (clopidogrel 75 mg + rivaroxaban 15 mg).*
